# AAA+ Machines of Protein Destruction in Mycobacteria

**DOI:** 10.3389/fmolb.2017.00049

**Published:** 2017-07-19

**Authors:** Adam Atlas Thomas, David A. Dougan

**Affiliations:** Department of Biochemistry and Genetics, La Trobe Institute for Molecular Science, La Trobe University Melbourne, VIC, Australia

**Keywords:** AAA+ protease complexes, protein degradation, Mycobacterium, novel drug targets, proteasome

## Abstract

The bacterial cytosol is a complex mixture of macromolecules (proteins, DNA, and RNA), which collectively are responsible for an enormous array of cellular tasks. Proteins are central to most, if not all, of these tasks and as such their maintenance (commonly referred to as protein homeostasis or proteostasis) is vital for cell survival during normal and stressful conditions. The two key aspects of protein homeostasis are, (i) the correct folding and assembly of proteins (coupled with their delivery to the correct cellular location) and (ii) the timely removal of unwanted or damaged proteins from the cell, which are performed by molecular chaperones and proteases, respectively. A major class of proteins that contribute to both of these tasks are the AAA+ (ATPases associated with a variety of cellular activities) protein superfamily. Although much is known about the structure of these machines and how they function in the model Gram-negative bacterium *Escherichia coli*, we are only just beginning to discover the molecular details of these machines and how they function in mycobacteria. Here we review the different AAA+ machines, that contribute to proteostasis in mycobacteria. Primarily we will focus on the recent advances in the structure and function of AAA+ proteases, the substrates they recognize and the cellular pathways they control. Finally, we will discuss the recent developments related to these machines as novel drug targets.

## Tuberculosis

Tuberculosis (TB) is a devastating disease that currently affects approximately one third of the world's population. Each year TB is responsible for over 1 million deaths with almost 10 million new cases being diagnosed. The disease is caused by a single pathogen—*Mycobacterium tuberculosis* (*Mtb*) and although the disease is eminently curable, the inappropriate administration of drugs has led to the emergence of several drug resistant strains, which are increasingly more difficult to eradicate. Most recently, a totally drug-resistant (TDR) strain of *Mtb* has emerged, which as the name suggests is resistant to all available drugs for the treatment of TB. Hence, there is an urgent need to develop new drugs that target novel pathways within these resistant strains. An emerging approach is the targeting of proteases.

## AAA+ proteases in mycobacteria

**Figure 1:**
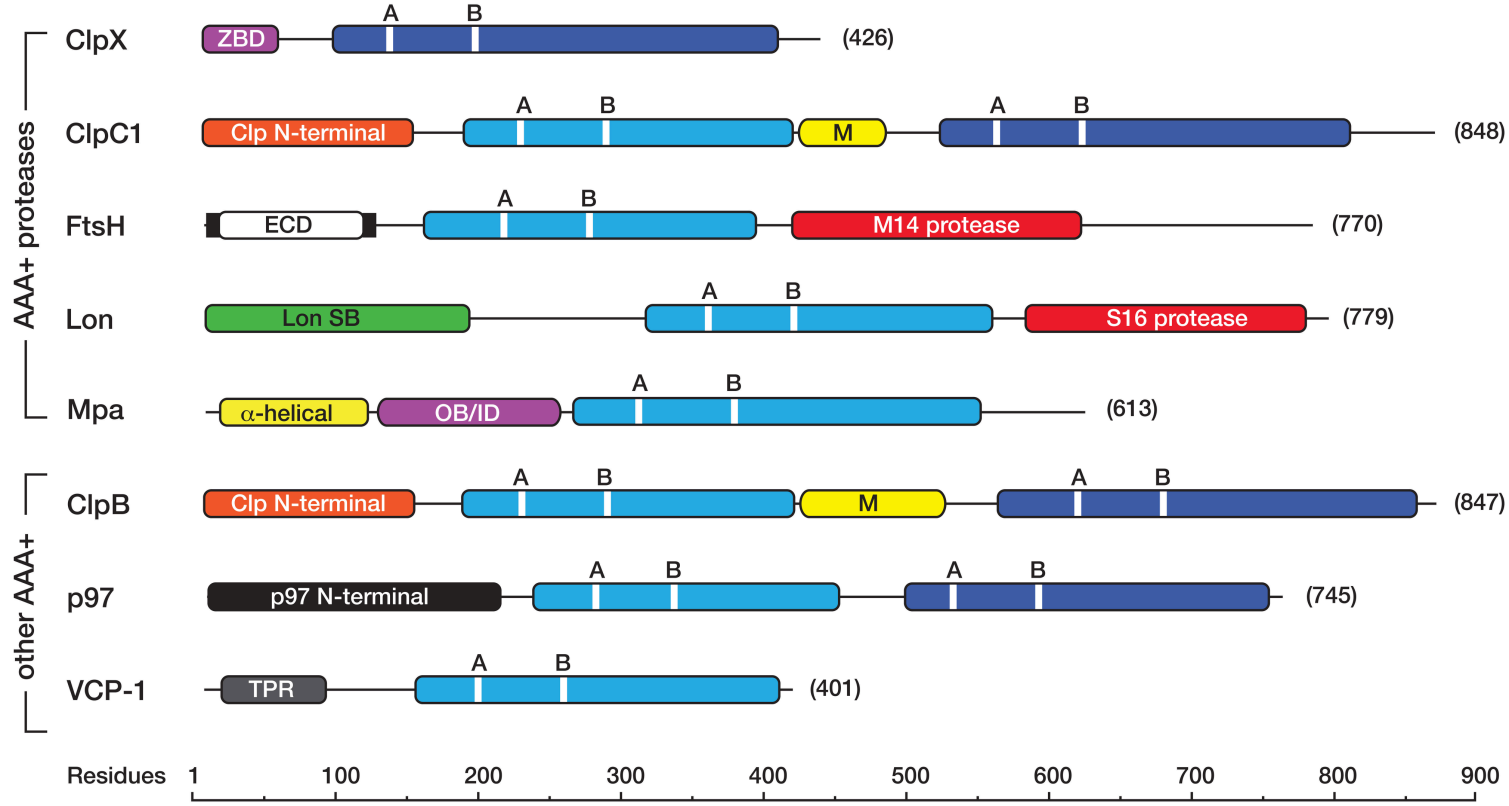
Linear cartoon of the different AAA+ proteins in mycobacteria, illustrating the position of various domains and motifs. The AAA+ domains either belong to the classic (light blue) or HCLR (dark blue) clade. Each AAA+ domain contains a consensus sequence for ATP binding (GX4GKT/S, where X is any amino acid) and hydrolysis (hDD/E, where h is any hydrophobic amino acid) known as the Walker A (A), and Walker B (B) motifs, respectively. Most AAA+ proteins contain an unique accessory domain, such as the zinc-binding domain (ZBD, in pink) in ClpX, the Clp N-terminal domain (orange) in ClpC1 and ClpB, the Lon SB (substrate binding) domain (green) in Lon, the α-helical (yellow) and OB/ID (pink) domains in Mpa, the p97 N-terminal domain (black) in *Msm*0858 and the Tetratricopeptide (TPR)-like domain (gray) in VCP-1. ClpC1 and ClpB also contain a middle (M) domain (yellow) located between the first and second AAA+ domain. The membrane-bound AAA+ protein, FtsH contains two transmembrane domains (black bars) separated by an extracellular domain (ECD, in white) and a C-terminal metallopeptidase (M14 peptidase) domain (red) containing the consensus sequence (HEXGH). Lon contains an N-terminal substrate binding (Lon SB) domain a central AAA+ domain and a C-terminal serine (S16) peptidase domain (red) with the catalytic dyad (S, K). All cartoons are derived from the sequences for the following *M. smegmatis* proteins ClpX (A0R196), ClpC1 (A0R574), FtsH (A0R588), Lon (O31147), Mpa (A0QZ54), ClpB (A0QQF0), p97/*Msm*0858 (A0QQS4), VCP-1/*Msm*1854 (A0QTI2). Domains (and domain boundaries) were defined by InterPro (EMBL-EBI) as follows: AAA+ (IPR003593); C4-type Zinc finger (IPR010603); Clp N-terminal (IPR004176); UVR or M (IPR001943); Lon SB (substrate binding) (IPR003111); p97 N-terminal (IPR003338); p97 OB/ID (IPR032501); Tetratricopeptide (TPR)-like (IPR011990); S16 protease (IPR008269), M41 protease (IPR000642).

**Figure 2:**
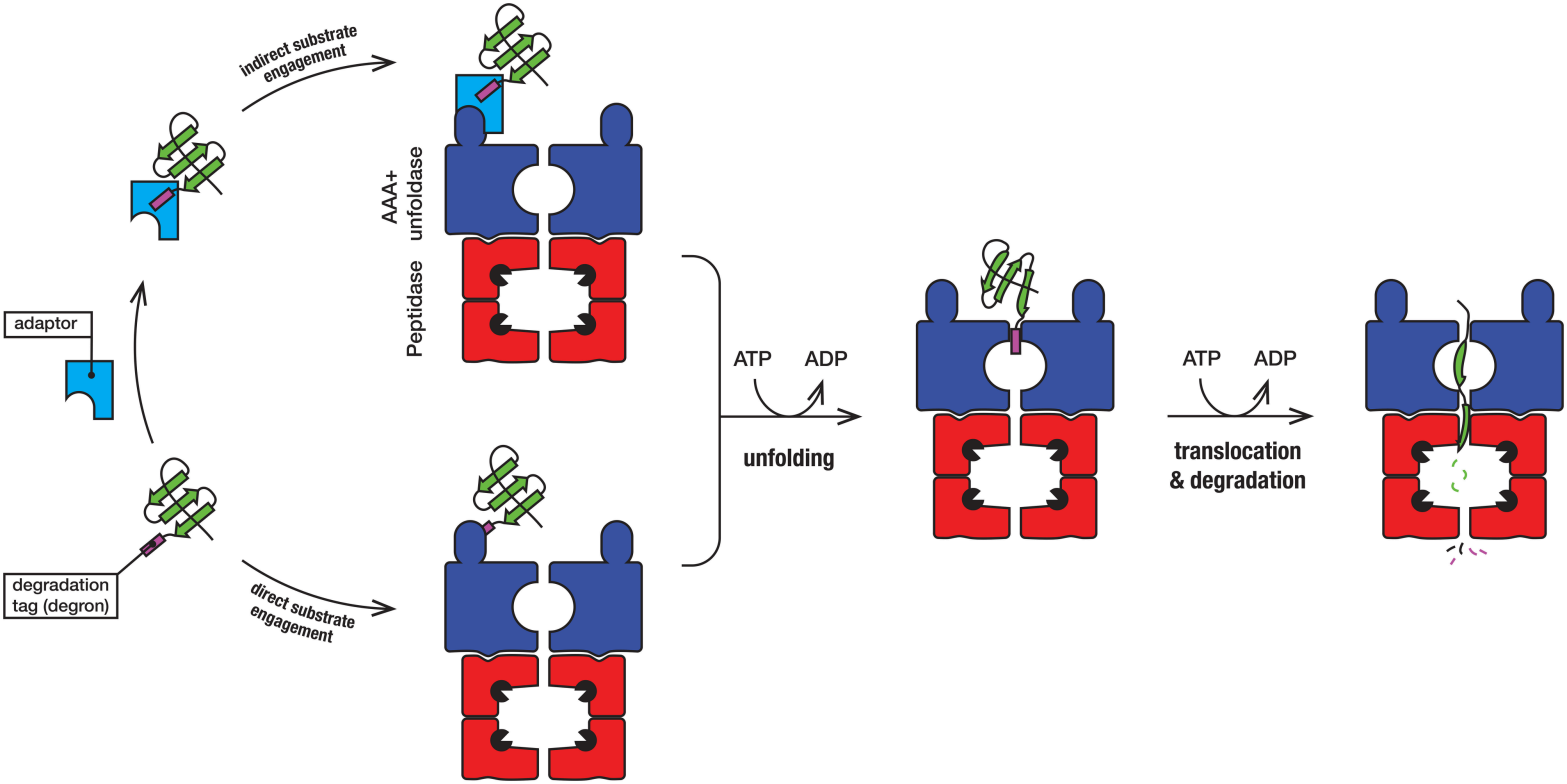
In the first step, the substrate (green) engages with the AAA+ unfoldase (blue) via the degradation tag (commonly referred to as a degron). The degron (purple) is generally located at the N- or C-terminal end of the substrate, although in some case it may be internal (and exposed following unfolding or dissociation of the protein from a complex). For direct recognition by the AAA+ unfoldase (blue), the degron is engaged either by a specialized accessory domain or by specific loops, located at the distal end of the machine. Following recognition of the degron, the substrate protein is unfolded by the ATP-dependent movement of axial pore loops. The unfolded substrate is then translocated into the associated peptidase (red), where the peptide bonds are hydrolyzed by the catalytic residues (black packman) into short peptides. The peptides are released, either through the axial pore or holes in the side walls that are created during the cycle of peptide hydrolysis.

Protein degradation is a fundamental cellular process that controls the irreversible removal of proteins from the cell. Given the definitive nature of this process, the machines that control protein turnover in the cell must be tightly regulated to prevent the unwanted turnover of normal cellular proteins. At the same time, these proteases need to permit, not only the broad recognition of damaged proteins, but also the precise recognition of specific regulatory proteins in a timely fashion. In bacteria, this is achieved by a collection of proteolytic machines (together with their cofactors), which mediate the explicit recognition of a diverse set of protein substrates. Not surprisingly, proteases have been identified as important drug candidates and the dysregulation of these machines has been demonstrated to kill both dormant and actively dividing cells (Brotz-Oesterhelt et al., 2005; Conlon et al., 2013). Mycobacteria such as *Mtb* [and *Mycobacterium smegmatis* (*Msm*), a close non-pathogenic relative of *Mtb*], are rod-shaped acid fast staining bacteria that retain characteristics of both Gram-positive and Gram-negative bacteria and as such they contain a somewhat unique composition of proteins. In mycobacteria, protein turnover in the cytosol is mediated by at least four different ATP-dependent machines (Figure 1), several of which are essential (Sassetti et al., 2003; Raju et al., 2014). Broadly speaking, these machines can be arranged into two groups, (i) the *bacterial-like* proteases [which include FtsH and Lon as well as the Casein lytic protein (Clp) proteases ClpC1P and ClpXP] and (ii) the *eukaryotic-like* proteasome. They are typically composed of two components—a barrel-shaped peptidase that is capped at one or both ends, by a ring-shaped unfoldase (Figure 2). Invariably the unfoldase component belongs to the AAA+ (ATPases associated with a variety of cellular activities) superfamily and as such they are commonly referred to as AAA+ proteases (Sauer and Baker, 2011; Gur et al., 2013). Although a few of these machines (e.g., FtsH and Lon) contain both components on a single polypeptide, most machines (e.g., ClpC1P, ClpXP, and Mpa-20S) contain each component on separate polypeptides. The steps in the degradation pathway of these machines are generally conserved (Figure 2). In the first step, the substrate is either directly engaged by the unfoldase, or indirectly engaged by an adaptor protein before it is delivered to the unfoldase. Regardless of the initial mode of contact, substrate engagement by the unfoldase is generally mediated by specialized accessory domains and/or specific loops, located at the distal end of the machine (Figure 2). Following this step, the substrate is translocated through the central pore of the unfoldase (in an ATP-dependent manner), into the proteolytic chamber of the associated peptidase where the substrate is cleaved into small peptide fragments. Interestingly, in some cases these peptidases are also activated for the energy-independent turnover of specific protein substrates, through the interaction with non-AAA+ components (Bai et al., 2016; Bolten et al., 2016). These nucleotide-independent components facilitate substrate entry into the proteolytic chamber by opening the gate into the peptidases, as such we refer to them as gated dock-and-activate (GDA) proteases. Although this group of proteases is not the focus of this review, we will discuss them briefly (see later).

## The Clp protease(s)

The Clp protease is a large multi-subunit complex composed of a barrel-shaped peptidase (ClpP) flanked on either or both ends by a hexameric AAA+ unfoldase (ClpX or ClpC1). Interestingly, in contrast to most bacteria, the Clp protease is essential in *Mtb*, not only for virulence but also for cell viability (Sassetti et al., 2003; Carroll et al., 2011; Raju et al., 2012). It is also essential for viability in *Msm*, indicating that beyond its role in virulence, the Clp protease plays a crucial role in “general” proteostasis. Consistently, the Clp protease is responsible for regulation of various stress responses in both *Mtb* (Barik et al., 2010; Raju et al., 2014) and *Msm* (Kim et al., 2009), as well as the turnover of incomplete translation products that have been co-translationally tagged with the SsrA sequence (Raju et al., 2012; Personne et al., 2013).

### Processing and activation of the peptidase (ClpP)

**Figure 3:**
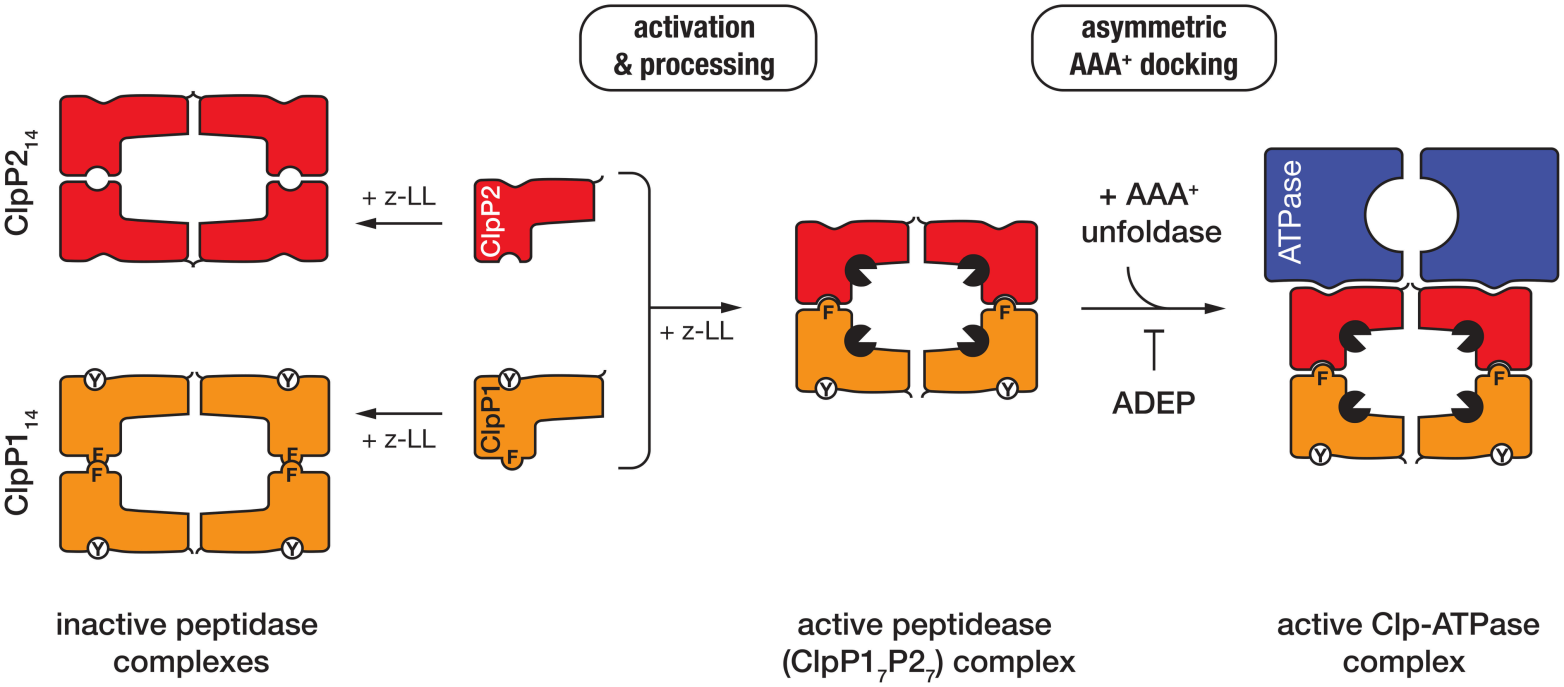
In the presence of the dipeptide activator (z-LL), ClpP1 (orange), and ClpP2 (red) form either homo- (left) or hetero-oligomeric complexes (middle). Activator binding is essential for propeptide processing of both ClpP proteins in *Mtb* (while only ClpP1 is processed in *Msm*). Hetero-oligomeric complexes are activated (black packman) through the complementary docking of Phe147 (F) of ClpP1, into a pocket on the handle of ClpP2. In contrast, homo-oligomeric complexes lack this complementary docking and are not active. The unfoldase (blue) docks only to a single face of the active peptidase (i.e., ClpP2) to generate an asymmetric machine. ADEP docks only to the hydrophobic pockets of ClpP2 and as such prevents docking of the unfoldase component.

The peptidase component of the Clp protease—ClpP, is composed of 14 subunits, arranged into two heptameric rings stacked back-to-back. The active site residues of ClpP are sequestered inside the barrel-shaped oligomer away from the cytosolic proteins. Entry into the catalytic chamber is restricted to a narrow entry portal at either end of the barrel. Although the overall architecture of these machines is broadly conserved (across most bacterial species), the composition and assembly of the ClpP complex from mycobacteria is atypical. In contrast to most bacteria, mycobacteria contain two ClpP homologs (ClpP1 and ClpP2), both of which form homo-heptameric ring-shaped oligomers. Although these homo-oligomers can assemble into both homo- and hetero-tetradecamers, only the hetero-oligomeric complexes (composed of a single ring of each subunit) exhibit catalytic activity *in vitro* (Akopian et al., 2012; Schmitz et al., 2014) (Figure 3). Unexpectedly, the *in vitro* activity of this complex was also dependent on the presence of a novel dipeptide activator—benzyloxycarbonyl-leucyl-leucine [z-LL] and each ring of the active complex displays unique specificity (Akopian et al., 2012; Personne et al., 2013; Li et al., 2016).

Similar to *E. coli* ClpP (*Ec*ClpP), both *Mtb* ClpPs (ClpP1 and ClpP2) are expressed as proproteins. However, in contrast to *Ec*ClpP (in which the propeptide is auto-catalytically processed), the processing of both *Mtb* ClpPs, appears to occur in a sequential fashion, possibly via an *in trans* mechanism. Specifically, the propeptide of *Mtb*ClpP2 is initially processed by the active sites of *Mtb*ClpP1, before propeptide cleavage of *Mtb*ClpP1 can occur (Leodolter et al., 2015). Currently however, it remains unclear if cleavage of the *Mtb*ClpP1 propeptide also occurs *in trans* (via the active site residues of *Mtb*ClpP2) or simply requires interaction with “active” processed *Mtb*ClpP2 for autocatalytic processing. Consistent with the *in trans* processing observed for the *Mtb*ClpP1P2 complex, *Msm*ClpP2 also appears to be processed by the catalytic residues of *Msm*ClpP1, however the precise location of this processing event remains uncertain (Akopian et al., 2012). Likewise, it remains unclear if *Msm*ClpP1 contains a propeptide, as the *in vitro* processing of *Msm*ClpP1 has yet to be observed (Benaroudj et al., 2011; Akopian et al., 2012; Leodolter et al., 2015). Additional experiments are still required to fully understand the mechanism of processing and activation of this complex.

Recently the crystal structure of *Mtb*ClpP1P2, in complex with an alternative activator (z-IL) and the ClpP-specific dysregulator (acyldepsipeptide, ADEP, see later) was solved to 3.2 Å (Schmitz et al., 2014). This structure (in comparison to the inactive *Mtb*ClpP1P1 complex) provided a detailed understanding of how the hetero-oligomeric complex is assembled and activated (Ingvarsson et al., 2007; Schmitz et al., 2014). Notably, the *Mtb*ClpP1P2 structure is formed by a single homo-oligomeric ring of each subunit, the shape (and dimensions) of which is significantly different to that of the inactive ClpP1 homooligomer (Ingvarsson et al., 2007; Schmitz et al., 2014). The active complex, forms an “extended” conformation (~93 Å high × 96 Å wide) which is stabilized by the complementary docking of an aromatic side-chain (Phe147) on the ClpP1 handle, into a pocket on the handle of ClpP2 (Schmitz et al., 2014). This docking, switches the catalytic residues of both components into the active conformation. By contrast the ClpP1 tetradecamer, which lacks this complementary handle recognition, is compressed (~10 Å flatter and wider) and as a result the catalytic residues are distorted from their active conformation (Figure 3). This structure also revealed that the peptide “activator” was bound in the substrate binding pocket (of all 14 subunits), albeit in the reverse orientation of a *bona fide* substrate (Schmitz et al., 2014). This provided a structural explanation for why high concentrations of the activator inhibit protease activity (Akopian et al., 2012; Famulla et al., 2016). Significantly, the *Mtb*ClpP1P2 structure also established that the ClpP-dysregulator, (ADEP) only interacts with a single ring of the complex (namely *Mtb*ClpP2). Interestingly, despite docking to a single ring, ADEP triggered pore opening of both rings of the complex (the *cis* ring to to 25 Å and the *trans* ring to 30 Å). This simultaneous opening of both pores is thought, not only, to facilitate translocation of substrates into the chamber, but also likely to promote the efficient egress of the cleaved peptides (Figure 3). Consistent with the asymmetric docking of ADEP to the *Mtb*ClpP1P2 complex, Weber-Ban and colleagues recently demonstrated that both unfoldase components (*Mtb*ClpC1 and *Mtb*ClpX) also only dock to *Mtb*ClpP2, generating a truly asymmetric Clp-ATPase complex (Leodolter et al., 2015). This asymmetric docking of both unfoldase components appears to be driven by the presence of an additional Tyr residue within the hydrophobic pocket of ClpP1, which prevents unfoldase-docking to this component. The reason for this asymmetry is currently unclear, although one possibility is that an alternative component docks to the “shallow” hydrophobic pocket of ClpP1, thereby expanding the substrate repertoire of the peptidase. Consistent with this idea, an ATP-independent activator of the ClpP protease has recently been identified in *Arabidopsis thaliana* (Kim et al., 2015).

Although the Clp protease is essential in mycobacteria, only a handful of substrates have been identified. The currently known Clp protease substrates include aborted translation products tagged with the SsrA sequence, the anti-sigma factor RseA, and several transcription factors, WhiB1, CarD, and ClgR (Barik et al., 2010; Raju et al., 2012, 2014; Yamada and Dick, 2017). Of the known substrates, only RseA has been extensively characterized. In this case, phosphorylation of RseA (on Thr39) triggers its specific recognition by the unfoldase, *Mtb*ClpC1 (Barik et al., 2010). This phosphorylation-dependent recognition of RseA is reminiscent of substrate recognition by ClpC from *Bacillus subtilis* (*Bs*ClpC), which is also responsible for the recognition of phosphoproteins, albeit in this case proteins that are phosphorylated on Arg residues (Kirstein et al., 2005; Fuhrmann et al., 2009; Trentini et al., 2016). Interestingly, both *Bs*ClpC and *Mtb*ClpC1 also recognize the phosphoprotein casein, which is often used as a model unfolded protein. However, it currently remains to be seen if *Mtb*ClpC1 specifically recognizes phosphorylated Thr residues (i.e., pThr) or whether phosphorylation simply triggers a conformation change in the substrate. Likewise, it remains to be determined if misfolded proteins are generally targeted for degradation by ClpC1 *in vivo* or whether this role falls to alternative AAA+ proteases in mycobacteria. In contrast to RseA (which contains an internal phosphorylation-induced motif), the remaining Clp protease substrates contain a C-terminal degradation motif (degron). Based on the similarity of the C-terminal sequence of each substrate to known *Ec*ClpX substrates (Flynn et al., 2003), we speculate that these substrates (with the exception of WhiB1) are likely to be recognized by the unfoldase ClpX. Significantly, the turnover of both transcription factors (WhiB1 and ClgR) is essential for *Mtb* viability.

### Potential adaptor proteins of ClpC1 and ClpX

As illustrated in Figure 2, substrate recognition by AAA+ proteases is generally mediated by the AAA+ unfoldase component, however in some case this may be facilitated by an adaptor protein (Kirstein et al., 2009b; Kuhlmann and Chien, 2017). Adaptor proteins are generally unrelated in sequence or structure. Invariably they recognize a specific substrate (or class of substrates), which is delivered to their cognate unfoldase, by docking to an accessory domain of the unfoldase. In some cases, adaptor docking not only delivers the substrate to the unfoldase, but also activates the unfoldase, for substrate recognition (Kirstein et al., 2005; Rivera-Rivera et al., 2014). In the case of ClpX, most known adaptor proteins dock onto the N-terminal Zinc binding domain (ZBD). Despite the conserved nature of this accessory domain in ClpX, across a broad range of bacterial species, a ClpX adaptor protein has yet to be identified (either biochemically or bioinformatically) in mycobacteria. Nevertheless, given that most of the ClpX adaptor proteins that have been identified in bacteria are associated with specialized functions of that species, we speculate that mycobacteria have evolved a unique ClpX adaptor (or set of adaptors) that are unrelated to the currently known ClpX adaptors. In contrast to ClpX, mycobacteria are predicted to contain at least one ClpC1-specific adaptor protein—ClpS. In *E. coli*, ClpS is essential for the recognition of a specialized class of protein substrates that contain a destabilizing residue (i.e., Leu, Phe, Tyr, or Trp) at their N-terminus (Dougan et al., 2002; Erbse et al., 2006; Schuenemann et al., 2009). These proteins are degraded either by ClpAP (in Gram positive bacteria) or ClpCP (in cyanobacteria) via a conserved degradation pathway known as the N-end rule pathway (Varshavsky, 2011). Although most of the substrate binding residues in mycobacterial ClpS are conserved with *E. coli* ClpS (*Ec*ClpS), some residues within the substrate binding pocket have been replaced and hence it will be interesting to determine the physiological role of mycobacterial ClpS and whether this putative adaptor protein exhibits an altered specificity in comparison to *Ec*ClpS.

## FtsH

FtsH is an 85 kDa, membrane bound Zn metalloprotease. It is composed of three discrete domains, a extracytoplasmic domain (ECD) which is flanked on either side by a transmembrane (TM) region (Figure 1). The TM regions tethered the protein to the inner membrane, placing the ECD in the “pseudoperiplasmic” space (Hett and Rubin, 2008). The remaining domains (the AAA+ domain and M14 peptidase domain) are located within the cytosol. To date the function of FtsH is poorly understood in mycobacteria, and currently it is unclear if *ftsH* is indeed an essential gene (Lamichhane et al., 2003; Sassetti et al., 2003). Nevertheless, based on complementation experiments in an *E. coli ftsH* mutant strain, it appears that *Mtb*FtsH shares an overlapping substrate specificity with *Ec*FtsH, as it can recognize both cytosolic proteins (such as transcription factors and SsrA-tagged proteins) as well as membrane bound proteins (such as SecY). Hence *Mtb*FtsH is proposed to play a role in general protein quality control, stress response pathways, and protein secretion (Srinivasan et al., 2006). It is also proposed to play a crucial role in cell survival as it is reported to be transcriptionally upregulated in response to agents that produce reactive oxygen intermediates and reactive nitrogen intermediates (RNIs) in macrophages (Kiran et al., 2009).

## Lon

Lon is a broadly conserved AAA+ protease, which although absent from *Mtb* is present in several mycobacterial species, including *Msm* (Knipfer et al., 1999). In *Msm*, Lon is an 84 kDa protein composed of three domains, an N-terminal domain, which is generally required for substrate engagement, a central AAA+ domain and a C-terminal S16 peptidase domain (Figure 1). The physiological role of mycobacterial Lon is currently unknown and to date no physiological substrates have been identified. Despite the lack of physiological substrates available, *Msm*Lon like many Lon homologs can recognize and degrade the model unfolded protein, casein (Rudyak and Shrader, 2000; Bezawork-Geleta et al., 2015). Based, largely on the identification of casein as a model substrate, *Msm*Lon is predicted to be linked to the removal of unwanted misfolded proteins from the cell. Interestingly in *E. coli*, Lon also plays a crucial role in the regulation of persistence, through the activation of several Toxin-Antitoxin (TA) systems (Maisonneuve et al., 2013). Although *Msm* only contains a few TA systems, *Msm*Lon is expected to play a similar role to its *E. coli* counterpart. Surprisingly *Mtb* lacks Lon, but contains almost 100 TA systems (Sala et al., 2014). Hence it will be intriguing to determine how these different TA systems are activated in *Mtb* and which, if any, of the known AAA+ proteases contribute to this process. Nevertheless, the activity of *Msm*Lon appears to be highly regulated, as *Msm*Lon in addition to its catalytic peptidase site also contains two allosteric polypeptide binding sites (Rudyak and Shrader, 2000). Based on a series of *in vitro* experiments, it appears that the activity of *Msm*Lon is linked to its oligomerization, however in contrast to most AAA+ proteins, the oligomerization of *Msm*Lon is proposed to be mediated, not by ATP levels, but rather by the concentration of Mg^2+^ and the level of “unfolded” protein. These findings suggests that *in vivo* activity of Lon is tightly controlled by the presence of available substrate (Rudyak et al., 2001).

## The Pup-proteasome system (PPS)

In addition to the bacterial-like proteases, mycobacteria also contain an additional protease that shares similarity with the eukaryotic 26S proteasome. Similar to its eukaryotic counterpart [which is responsible for the degradation of proteins that have been marked for destruction with ubiquitin (Ub)], the mycobacterial proteasome is responsible for the recognition and removal of proteins that have been tagged by a protein called Pup (Prokaryotic Ub-like Protein). The conjugation of Pup to a substrate protein is referred to as Pupylation (see below) and collectively the proteolytic system is referred to as the Pup Proteasome System (PPS). Remarkably, despite the obvious functional similarities between Pup and Ub, the proteins are not conserved nor are the steps involved in their conjugation to substrates. Significantly, the PPS plays a crucial role in *Mtb* persistence and virulence by protecting cells from Nitric oxide and other RNIs that are produced by host macrophages during infection (Darwin et al., 2003).

### Prokaryotic ubiquitin (Ub)-like protein (Pup) and pupylation

Pup is a small (64 residue) unstructured protein (Chen et al., 2009) that although unrelated to Ub in sequence and structure, shares a common function with Ub. It is expressed in an inactive form [sometimes referred to as Pup(Q)] that contains a C-terminal Gln. The activation of Pup(Q) is mediated by an enzyme called Dop (Deamidase Of Pup), which involves the deamidation of the C-terminal Gln (to Glu) to generate Pup(E) (Striebel et al., 2009; Burns et al., 2010a). Once activated, the C-terminus of Pup(E) is first phosphorylated by PafA (Proteasome Accessory Factor A) through the hydrolysis of ATP, then attached to a substrate Lys residue by PafA, via the formation of an isopeptide bond between the C-terminal γ-carboxylate of Pup(E) and the ε-amino group of a Lys residue on the substrate in a process known as pupylation (Pearce et al., 2008; Forer et al., 2013).

Pupylation is involved in a variety of different physiological roles. In pathogenic bacteria such as *Mtb*, it plays an important role not only in virulence, protecting the cell from nitrosative stress (Darwin et al., 2003) but also in copper homeostasis (Shi et al., 2014), while in *Msm* it has been implicated in amino acid recycling under nutrient starvation conditions (Elharar et al., 2014). Given the diverse range of physiological roles, it is not surprising that the molecular targets of pupylation also vary from species to species. Although the target of pupylation, responsible for regulating copper homoestasis in *Mtb* has yet to be identified, Darwin and colleagues recently identified Log (Lonely guy) as the molecular target of pupylation that is responsible for protection of *Mtb* against nitrosative stress (Samanovic et al., 2015). Log is responsible for synthesis of the hormone, cytokinin. In *Mtb*, Log accumulates in cells lacking a component of the PPS, triggering the overproduction of cytokinin, which results in the toxic accumulation of aldehydes (breakdown products of cytokinin). In contrast to the regulation of nitrosative stress in *Mtb*, which involves the pupylation of a single target, *Msm* cells pupylate many targets in their response to nutrient starvation (Elharar et al., 2014). Indeed, Gur and colleagues demonstrated that high molecular weight proteins were preferentially targeted for pupylation under nutrient starvation conditions, and proposed that the turnover of these proteins was more efficient for amino acid recycling, than that of low molecular weight proteins. Consistently, the same group have recently demonstrated that during starvation, the opposing size preference of Dop and PafA, supports the preferential pupylation of high molecular weight proteins (Elharar et al., 2016). Pupylation has also recently been proposed to regulate iron homeostasis in *Corynebacterium glutamicum*. Interestingly, this bacterial species lacks both subunits of the 20S core particle (CP), and hence it is proposed that the pupylation-mediated regulation of iron homeostasis is independent of protein turnover. In this case, the target of pupylation is a single protein—ferritin, which is pupylated at Lys78. Ferritin is an iron storage protein which forms a cage composed of 24 identical subunits that encapsulates ~4,500 iron atoms (Andrews, 2010). Under iron limitation conditions, normal cells access this stored iron through disassembly of the ferritin cage, which is mediated by ARC (a homolog of Mpa, see below). In contrast, in cells lacking components of the pupylation machinery, ARC is unable to disassemble the ferritin complex and as a result these cells are unable to access the stored iron and hence exhibit strong growth defects under iron limitation conditions (Kuberl et al., 2016). In addition to these reports, several proteomic studies have identified that over 100 different proteins are pupylated (Festa et al., 2010; Poulsen et al., 2010; Watrous et al., 2010). However, whether each pupylated protein regulates a specific response or whether the complete set of pupylated proteins serve a collective purpose is yet to be defined. Nevertheless, these proteomic studies demonstrated that pupylation is a selective process, as only specific exposed Lys residues were modified. This suggests that PafA, likely displays some degree of substrate specificity beyond the target Lys residue and hence residues surrounding the target Lys may modulate interaction with PafA. Alternatively, it may suggest, that mycobacteria contain an additional factor that modulates substrate recognition by PafA.

### The mycobacterial proteasome

**Figure 4:**
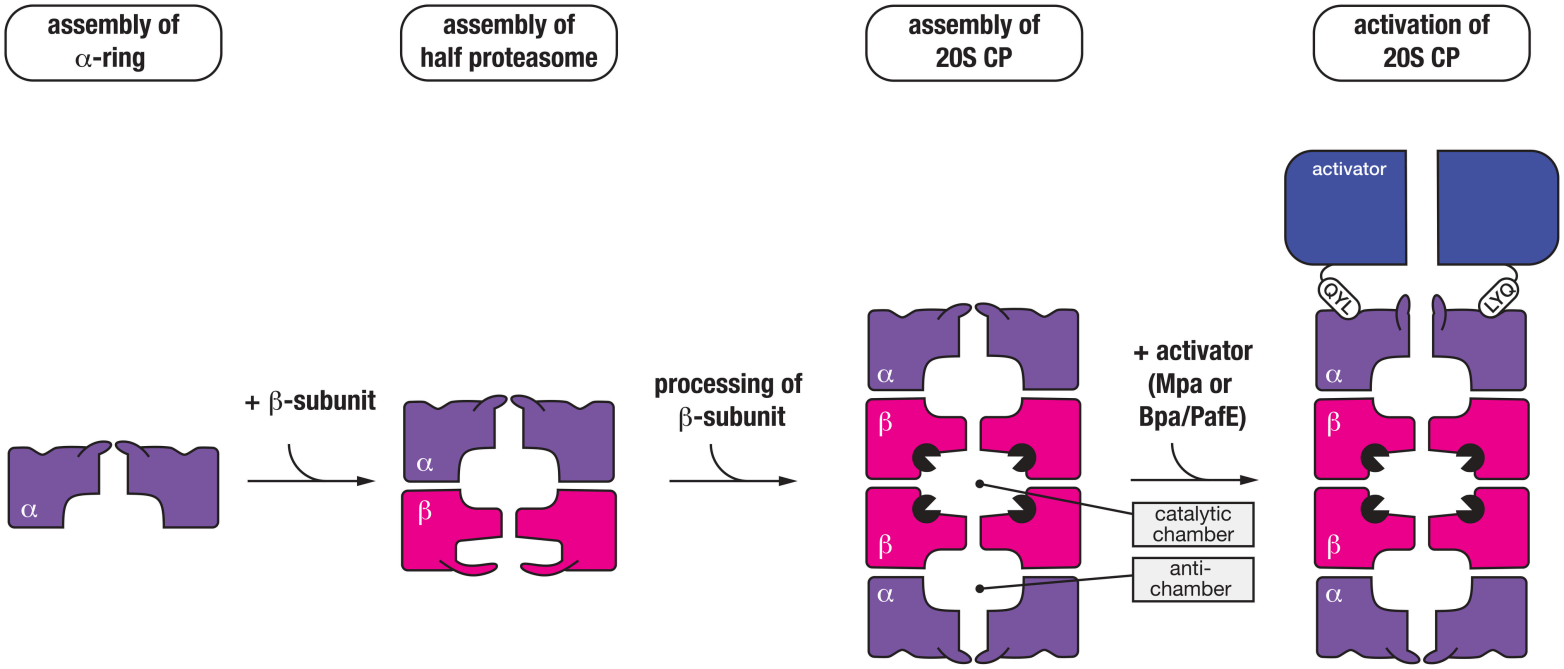
Seven α-subunits (purple) first assemble into a heptameric ring (α-ring), which is used as a template to form a half-proteasome, by assembly of the β-subunits into a heptameric ring (on the α-ring template). Next, two half-proteasomes assemble, triggering removal of the N-terminal propeptide of the β-subunits and activation of the 20S CP. Finally, the C-terminal QYL motif of an activator (blue) such as Mpa or PafE/Bpa docks into a hydrophobic pocket on the α-ring of the proteasome, which triggers “gate-opening” of the N-terminal peptides thereby allowing access of substrates into the catalytic chamber of the protease.

The mycobacterial proteasome is a multi-subunit machine composed of two components, a central peptidase component called the 20S CP which is flanked at either or both ends by a ring-shaped activator (Figure 4). The 20S CP is composed of four stacked heptameric rings; two outer rings composed of seven identical α-subunits (PrcA) and two inner rings composed of seven identical β-subunits (PrcB) (Hu et al., 2006; Lin et al., 2006). The β-subunits are catalytically active and hence form the central proteolytic chamber, while the α-subunits are catalytically inactive form a cap for the protease that interacts with different regulatory components. Assembly and maturation of the 20S CP is a multistep process. First the α_7_ ring is formed, which creates a template for the folding and assembly of the β_7_ ring (Lin et al., 2006). This complex (α_7_β_7_), termed the half-proteasome, assembles (via the β_7_ interface) to generate a full proteasome. In contrast to the eukaryotic proteasome, it appears that the mycobacterial 20S CP does not require additional factors for assembly (Bai et al., 2017). Following assembly of the full-proteasome, the β-subunit propeptide is autocatalytically processed, exposing a new N-terminal residue (Thr56), which forms the catalytic nucleophile of the mature complex (Zuhl et al., 1997; Witt et al., 2006) (Figure 4). Like ClpP, the catalytic residues of the 20S CP are sequestered inside the proteolytic chamber of the mature complex, and access to this chamber is restricted by a narrow entry portal (~10 Å in diameter) at either end of the barrel. This entry portal is formed by the N-terminal residues of the α-subunits and opening of the portal (to gain access to the proteolytic chamber) is controlled by the activator binding which regulates movement of the N-terminal residues of the α-subunits (Lin et al., 2006). To date two proteasomal activators have been identified in mycobacteria; an ATP-dependent activator called Mpa (Mycobacterial proteasome ATPase) (Darwin et al., 2005) and a nucleotide-independent activator known as PafE (Proteasome accessory factor E) or Bpa (Bacterial proteasome activator) (Delley et al., 2014; Jastrab et al., 2015). Although both activators use a conserved mechanism to regulate gate-opening, they each recognize specific types of substrates and as such control distinct degradation pathways in mycobacteria.

### ATP-dependent proteasome activator—Mpa

**Figure 5:**
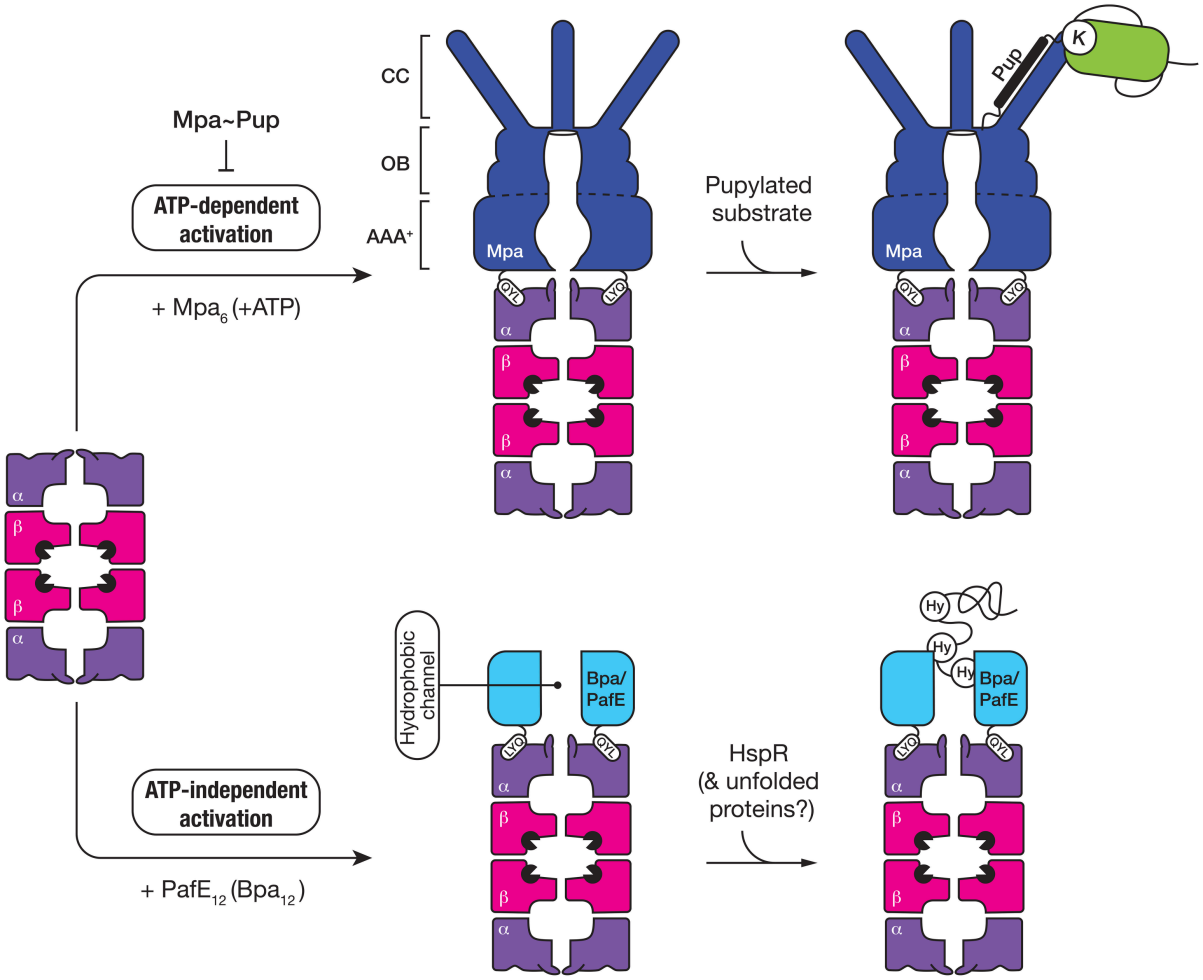
The 20S CP interacts with two different activators, both of which contain a QYL motif at the C-terminus to trigger “gate-opening” of the α-ring of the proteasome. Mpa (dark blue) is an ATP-dependent activator of the 20S CP (top panel). The ring-shaped hexamer is composed of three domains, a coiled-coil (CC) domain for interaction with pupylated substrates, an oligosaccharide/oligonucleotide-binding (OB) domain which stabilizes the hexamer and an AAA+ domain which uses the hydrolysis of ATP to drive unfolding of the pupylated substrate. The second activator (Bpa/PafE) is an ATP-independent dodecamer (light blue), which triggers “gate-opening” of the α-ring pore, by docking into the hydrophobic pockets on the surface of the α-ring. The ring-shaped dodecamer contains a wide (~40 Å) hydrophobic channel, which is proposed to interact with hydrophobic (Hy) residues that are exposed in proteins such as HspR (heat-shock protein R) and model unfolded proteins.

Mpa (the ATP-dependent activator of the proteasome) is responsible for the specific recognition of protein substrates that have been tagged with Pup. It is a 68 kDa protein composed of four distinct regions (Figure 5); an N-terminal α-helical domain (for interaction with Pup) and a C-terminal tail bearing the tripeptide motif, QYL (for docking to, and activation of the 20S CP) (Pearce et al., 2006), which are separated by an AAA+ domain and an interdomain region composed of two oligosaccharide/oligonucleotide-binding (OB) subdomains (OB1 and OB2). Although the AAA+ domain is directly responsible for ATP-binding and hence enzyme activity and the oligomerisation of Mpa, the interdomain region is also believed to promote assembly and stability of the Mpa oligomer as this region alone can form a hexamer in the absence of nucleotide (Wang et al., 2009, 2010). Once assembled into a hexamer, each pair of N-terminal α-helices (from adjacent subunits) associates to form a coiled-coil (CC). These CC structures protrude from the hexameric-ring like tentacles (Figure 5) and are directly responsible for the recognition of Pup (Striebel et al., 2010). Although each tentacle contains two Pup binding sites (one on each face), it appears that Pup only binds to the inner face of a single tentacle within the hexamer (Sutter et al., 2010; Wang et al., 2010). The interaction (between Pup and Mpa) is mediated by central region of Pup (residues 21–51), and docking to the tentacle occurs in an anti-parallel manner. This orientation of Pup, ensures that the unstructured N-terminus of Pup is directed toward the pore of Mpa, where it engages with the pore to initiate translocation of the substrate in an ATP-dependent fashion (Wang et al., 2009). Consistent with this idea, deletion of the N-terminal residues of Pup specifically prevented the *in vitro* turnover of pupylated substrates (Burns et al., 2010b; Striebel et al., 2010). Currently however, the fate of conjugated Pup is unclear, some evidence suggests that Pup, in contrast to Ub, is degraded together with the substrate (Striebel et al., 2010) while other evidence supports the idea that Pup is removed from the substrate, by Dop, before the pupylated substrate is degraded (Burns et al., 2010a; Cerda-Maira et al., 2010; Imkamp et al., 2010). The interaction with the 20S CP is mediated by the C-terminal tripeptide motif (QYL), which docks into a hydrophobic pocket on the α-ring. However, this motif is normally occluded by a β-grasp domain located within the C-terminal region of Mpa, which prevents efficient docking of the ATPase component to the 20S CP (Wu et al., 2017). As such, it has been proposed that additional factors may facilitate robust interaction between the ATPase and the protease. Interestingly, a single Lys residue near the C-terminus of Mpa is targeted by pupylation, which inhibits its ability not only to assemble, but also to dock to the 20S CP (Delley et al., 2012). Therefore, the pupylation of Mpa appears to serve as a mechanism to reversibly regulate the proteasome mediated degradation of pupylated substrates, which may play an important role in controlling the turnover of pupylated substrates.

### ATP-independent proteasome activator—Bpa/PafE

The first evidence for an additional proteasomal activator in mycobacteria came from comparison of the growth phenotypes of strains lacking different components of the proteasome, either *mpa* or *prcBA* (Darwin et al., 2003). The dramatic difference observed in the phenotypes displayed by these strains suggested that the 20S CP might be involved in the turnover of a separate class of substrate, likely through an additional activator. Recently two groups, independently identified a single novel activator of the proteasome—PafE/Bpa, which facilitates the ATP-independent turnover of the model unfolded substrate, β-casein (Delley et al., 2014; Jastrab et al., 2015). Like Mpa, PafE/Bpa contains the C-terminal motif (QYL), which is essential for its interaction with the hydrophobic pocket of the α-ring and activation of the proteasome (Figure 5). It also forms a ring-shaped complex, however in contrast to Mpa this complex is composed of 12 subunits which form a very large channel (~40 Å in diameter) that is lined with hydrophobic residues (Bai et al., 2016; Bolten et al., 2016). Although the mechanism of substrate recognition and release is not fully understood, it is proposed that the hydrophobic channel of PafE/Bpa interact with exposed hydrophobic residues in unfolded proteins. To date, the only physiological substrate to be identified is the heat shock protein repressor (HspR) (Jastrab et al., 2015).

## Other AAA+ proteins involved in mycobacterial proteostasis

In addition to the known AAA+ proteases in mycobacteria, three other AAA+ proteins are either known or predicted (based on annotated function/sequence homology) to play a role in proteostasis (Figure 1). They are ClpB, *Msm*0858/Rv0435c and Valosin containing protein-1 (VCP-1, also incorrectly annotated as Cdc48). VCP-1 (*Msm*1854) is a 43 kDa protein of unknown function. It contains a C-terminal AAA+ domain and an N-terminal Tetratrico peptide repeat (TPR)-like helical domain. Although the VCP-1 gene is only distributed in a limited number of Actinobacterial species (including *Msm*), it is invariably located in a putative operon, together with another gene of unknown function (MSMEG_1855). MSMEG_1855 encodes a membrane bound TPR-containing protein, which shares homology with *B. subtilis* BofA—a regulator of sporulation transcription factor, Sigma K (Zhou and Kroos, 2004). Therefore, we propose that VCP-1 (together with MSMEG_1855) is tethered to the inner membrane, and speculate that this complex regulates activation of a signal transduction pathway in mycobacteria.

*Msm*0858/Rv0435c (known as p97 in mammals or Cdc48 in yeast and plants) is a widely conserved 78 kDa protein, which is found in all kingdoms of life. In mammals, p97 plays a central role in the Ub proteasome system (UPS), where it not only interacts directly with ubiquitylated proteins to regulate their turnover, but also serves as a hub for the docking of numerous cofactors which help to mediate p97's many activities in the cell (for a detailed review of p97 function see Meyer and Weihl, 2014). Like mammalian p97, *Msm*0858 is composed of an N-terminal domain and two AAA+ domains. Interestingly, although the second AAA+ domain (D2) of *Msm*0858 exhibits a consensus sequence for both the Walker A and B motifs, critical residues in both motifs of the first AAA+ domain (D1) have been replaced (notably Thr in the Walker A motif is replaced with Val, while the first Asp in the Walker B motif is replaced with Ala). Despite these changes, both domains of *Msm*0858 displayed ATPase activity indicating that each domain can both bind and hydrolyze ATP (Unciuleac et al., 2016). Consistently, the recent crystal structure of *Msm*0858 revealed that the structures of the D1 and D2 domains of *Msm*0858 are highly similar to the equivalent domains in mammalian p97, with a root mean square deviation of 1.5 and 2.4 Å, respectively (Unciuleac et al., 2016). The structural similarity extends beyond the AAA+ domains of *Msm*0858, into its N-terminal domain, and despite this domain sharing only modest sequence similarity with mammalian p97 it shares significant structural similarity with its mammalian counterpart. In mammals, the N-terminal domain of p97 is an important docking platform for cofactor binding and hence the diverse activities of p97. This suggests that *Msm*0858 could serve a similar range of functions in mycobacteria, albeit using a distinct set of cofactors. Surprisingly, and in contrast to mammalian p97, *Msm*0858 was only observed to form a dimer in solution, however it remains to be seen if the lack of hexamer formation is due to the experimental conditions used, or alternatively it might indicate that a specific adaptor protein or cofactor is required for assembly or stabilization of the *Msm*0858 hexamer. Hence, it will be interesting to determine the oligomeric state of *Msm*0858 *in vivo*, and identify any factors that may modulate the activity of this highly conserved protein.

ClpB is a broadly conserved protein of ~ 92 kDa, that like ClpC1, is composed of two AAA+ domains which are separated by a middle domain (Figure 1). However, in contrast to ClpC1 (in which the M-domain is composed of two helices) the M-domain of ClpB is composed of four helices which form two coiled-coil motifs. In *Ec*ClpB, the M-domain serves as an important regulatory domain of the machine, as it represses the ATPase activity of the machine. It also serves as an important docking site for its co-chaperone DnaK. Collectively, ClpB and DnaK (together with its co-chaperones, DnaJ and GrpE) form a bi-chaperone network that is responsible for the reactivation of aggregated proteins. A similar role for mycobacterial ClpB was recently confirmed (Lupoli et al., 2016). Indeed, *Mtb*ClpB plays a crucial role in controlling the asymmetric distribution of irreversibly oxidized proteins (Vaubourgeix et al., 2015) and as such ClpB-deficient *Mtb* cells exhibit defects in recovery from stationary phase or exposure to antibiotics. Hence, ClpB might be a useful antibiotic target in the future, forcing cells to maintain their damaged proteome.

## AAA+ proteases as novel drug targets

Since the golden age of antibiotic discovery, very few new antibiotics have been bought to market and as a result, we are now seeing the rise of numerous antibiotic resistance bacteria. This includes, but is not limited to, the bacterial pathogen that is responsible for TB - *Mtb*. Indeed, there are currently three different strains of *Mtb*, each of which exhibits increasing resistance to available antibiotics. They are: multi drug resistant (MDR) *Mtb* which is resistant to the first line defense drugs isoniazid and rifampicin; extensively drug resistant (XDR) *Mtb* which is resistant to both first line defense drugs as well as to fluoroquinolones and at least one of the three injectable second line defense drugs, and totally drug resistant (TDR) *Mtb* which is resistant to all currently available drugs. As a consequence, there is an urgent need to develop new drugs that target novel pathways in these drug resistant strains of *Mtb*. Recently, several different components of the proteostasis network have been identified as promising novel drug targets in *Mtb*.

### Dysregulators of ClpP1P2 function: activators and inhibitors

**Figure 6:**
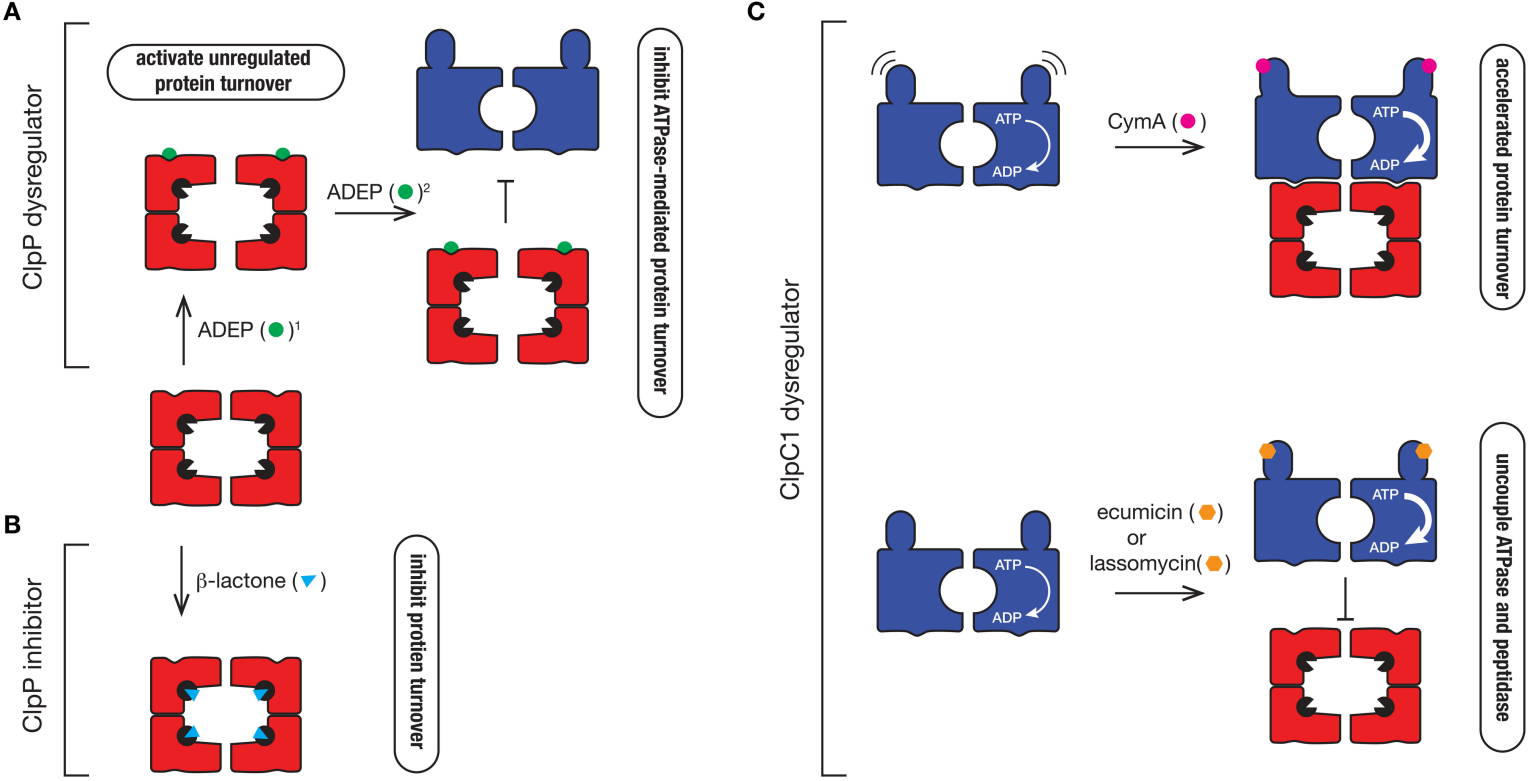
Mechanism of action of different Clp protease inhibitors and activators. **(A)** ClpP dysregulators such as ADEP (green circle) dock into the hydrophobic pocket of ClpP2, where they (1) activate the protease to trigger uncontrolled degradation of cellular proteins and (2) inhibit ATPase docking thereby preventing the regulated turnover of specific substrates that are delivered to the protease by the ATPase. **(B)** β-lactones (blue triangle) inhibit ClpP by inactivating the catalytic Ser (black packman) residue of the protease. **(C)** ClpC1 dyregulators such as CymA (pink circle), ecumicin (orange hexagon), or lassomycin (orange hexagon) bind to the N-terminal domain of ClpC1, accelerating its ATPase activity. In the case of CymA, docking to the N-terminal domain prevents movement of the domain, which triggers the accelerated turnover of proteins. In contrast, ecumicin and lassomycin uncouple ClpC1 from the peptidase, thereby preventing the regulated turnover of specific proteins.

In the Clp field, the interest in antibiotics was sparked by the identification of a novel class of antibiotics termed acyledepsipeptides (ADEPs) (Brotz-Oesterhelt et al., 2005). This class of antibiotic, was initially demonstrated to be effective against the Gram-positive bacterium, *B. subtilis* where it was shown to dysregulate the peptidase, ClpP. Specifically, ADEPs interact with the hydrophobic pocket of ClpP, triggering cell death via one of two suggested modes of action. The first mode-of-action is to activate the ClpP peptidase, by opening the gate into the catalytic chamber from ~10 Å to > 20 Å in diameter (Lee et al., 2010; Li et al., 2010). This results in the unregulated access of newly synthesized or unfolded proteins into the proteolytic chamber resulting in their indiscriminate degradation (Figure 6A). This mode-of-action activation appears to be crucial for ADEP-mediated killing of bacteria in which ClpP is not essential, such as *B. subtilis*. The second mode-of-action is to prevent docking of the partner ATPase (e.g., ClpC, ClpA, or ClpX), which inhibits the regulated turnover of specific substrates (Kirstein et al., 2009a). This mode-of-action appears to be critical in the ADEP-mediated killing of bacteria in which the unfoldase components are essential, such as *Mtb* (Famulla et al., 2016). Consistent with this idea, ADEPs only binds to one face of the ClpP1P2 complex—ClpP2, the face that is responsible for interaction with the ATPase component (Ollinger et al., 2012; Schmitz et al., 2014). Although these compounds are promising drug candidates, they currently exhibit poor drug-like qualities and are efficiently removed from the cell (Ollinger et al., 2012), hence additional development is required to improve their effectiveness *in vivo*.

Last year, the first non-peptide based activator of ClpP was identified from a screen of fungal and bacterial secondary metabolites (Lavey et al., 2016). In this case, the identified compound (Sclerotiamide) dysregulated *Ec*ClpP, by activating the ATPase-independent turnover of casein. Intriguingly, Sclerotiamide appears to be quite specific for *Ec*ClpP, as it was unable to dysregulate *Bs*ClpP, hence it will be interesting to see how and where this compound binds, and whether it will be able to activate other ClpP complexes such as the *Mtb*ClpP1P2 complex in the future.

In addition to the ClpP activators, several ClpP specific inhibitors have also been developed. The first group are the β-lactones (Figure 6B). These are suicide inhibitors that inactivate ClpP through the formation of an acyl-ester intermediate between the β-lactone ring (of the inhibitor) and the catalytic Ser of the peptidase which is much more stable than the intermediate formed between the substrate and the catalytic Ser during peptide bond catalysis (Bottcher and Sieber, 2008). In 2013 Sello and colleagues developed two β-lactone derivatives which killed *Mtb* cells (Compton et al., 2013). Interestingly, both β-lactones specifically target the ClpP2 component of the ClpP1P2 complex in *Mtb*, hence there is still potential for the development of ClpP1 inhibitors. Despite their effectiveness *in vivo*, most β-lactones exhibit poor stability in plasma and hence this will likely limit their future development (Weinandy et al., 2014).

The final inhibitor of ClpP1P2 was recently identified by Dick and colleagues from a whole-cell high throughput screen (Moreira et al., 2015). Interestingly, the compound they identified (bortezomib) is a known inhibitor of the human proteasome, which is currently being used in the treatment of multiple myeloma (under the commercial name, Velcade). Perhaps unsurprisingly, bortezomib has also been used in biochemical assays with the *Mtb* proteasome (Hu et al., 2006). Clearly the cross reactivity of bortezomib with the human proteasome represents a challenge for the future, although there are already promising signs that more specific ClpP1P2 inhibitors can be developed (Moreira et al., 2017).

### Dysregulators of ClpC1 function

Given the ATPase component(s) of the Clp protease are essential for viability, it is not surprising that dyregulators of these components also have antibacterial properties. Cyclomarin A (CymA) was the first identified dysregulator of the ClpC1 component of the Clp protease (Figure 6C). It is a cyclic non-ribosomal peptide that is produced by a marine bacterium (Renner et al., 1999). In 2011, CymA was identified as a potent antitubercular compound, which not only inhibited *Mtb* growth *in vitro*, but it also demonstrated bactericidal activity in human derived macrophages. Significantly, CymA also exhibited bactericidal activity against a panel of MDR strains of *Mtb* (Schmitt et al., 2011). Using a simple affinity chromatography approach, Schmitt and colleagues were able to show that CymA specifically bound to a single protein—ClpC1 (Schmitt et al., 2011). This binding appears to increase the ClpC1-medaited turnover of proteins in the cell and as such CymA was proposed to dysregulate ClpC1 function. Based on current structural data, CymA binds directly to the N-terminal domain of ClpC1 where it is proposed to alter the flexibility of this domain, thereby improving access of substrates to the pore of ClpC1 (Vasudevan et al., 2013). However, this mechanism of action has yet to be verified biochemically and hence the mode of CymA dysregulation remains uncertain. Intriguingly, the binding of CymA occurs near the docking site of adaptor proteins (MecA and ClpS) in equivalent systems (Kirstein et al., 2009b) and hence it is possible that CymA also modulates the docking of putative adaptor proteins in Mycobacteria.

Interestingly, the N-terminal domain of ClpC1 appears to be a common target of ClpC1 dysregulators, as two additional compounds were recently identified to bind to this region, ecumicin and lassomycin (Gavrish et al., 2014; Gao et al., 2015). Both compounds were identified from high-throughput screens; lassomycin from a screen using extracts of uncharacterized soil bacteria (Gavrish et al., 2014), while ecumicin was identified from a screen of actinomycetes extracts (Gao et al., 2015). Significantly, lassomycin not only inhibited the growth of wild type *Mtb* cells, but also exhibits potent antibacterial activity against MDR strains of *Mtb*, while ecumicin exhibited potent antibacterial activity against both actively dividing and dormant *Mtb* cells, as well as MDR and XDR strains of *Mtb*. Lassomycin is a ribosomally synthesized lasso-peptide that contains several Arg residues and hence is predicted to dock into an acidic patch on the N-domain of ClpC1. In contrast, ecumicin is a macrocyclic tridecapeptide composed of several non-cononical amino acids, which similar to CymA, is predicted to bind to in close proximity to a putative adaptor docking site (Gao et al., 2015; Jung et al., 2017). Interestingly, despite docking to different sites within the N-terminal domain, both compounds (lassomycin and ecumicin) stimulate the ATPase of ClpC1, but in contrast to CymA, they appear to uncouple the interaction between the ATPase and the peptidase, as they both inhibit the ClpC1-mediated turnover of the model unfolded protein, casein (Figure 6C). Currently however, it remains unclear if cell death results from the increased unfolding activity of ClpC1 or from the loss of ClpP1P2-mediated substrate turnover. Future efforts to determine the molecular mechanism of each compound are still required. This will likely be aided by structural studies of these compounds in complex with their target. Importantly, although further development of these compounds is still required to improve their pharmacokinetic properties, these compounds hold new hope in the battle against antibiotic resistant pathogens. It will also be interesting to see what else nature has provided in our ongoing battle against pathogenic microorganisms.

## Author contributions

AAT and DAD wrote and critically revised this work.

### Conflict of interest statement

The authors declare that the research was conducted in the absence of any commercial or financial relationships that could be construed as a potential conflict of interest. The reviewer CE and handling Editor declared their shared affiliation, and the handling Editor states that the process nevertheless met the standards of a fair and objective review.
